# LED Photo-polymerization, a Novel Strategy for Triggered Release Liposomes

**DOI:** 10.22037/ijpr.2019.112366.13712

**Published:** 2020

**Authors:** Afsoon Akbarzadeh, Pezhman Sasanpour, Hamid R. Moghimi

**Affiliations:** a *Department of Pharmaceutics and Nanotechnology, School of Pharmacy, Shahid Beheshti University of Medical Sciences, Tehran, Iran. *; b *Department of Medical Physics and Engineering, School of Medicine, Shahid Beheshti University of Medical Sciences, Tehran, Iran.*

**Keywords:** Liposome, LED light, Trigger release, Controlled release, Drug delivery

## Abstract

LED light is used for many medical and cosmetic applications such as phototherapy and skin rejuvenation. Such physical methods can be combined with drug therapy, such as LED-responsive drug delivery system, the subject of present investigation.

To perform this investigation, a nanoliposome composed of DPPC, DSPE-PEG2000, and DC_8,9_PC, was prepared as LED-sensitive systems. Calcein was loaded in the liposomes as a fluorescent probe for drug release studies. Different LED wavelengths (blue, green and red) were used for triggering release of calcein from nanoliposome. Indoor daylight, darkness, and sunlight were applied as controls.

Results showed that liposomes do not release their cargo in darkness, but they released it in response to indoor daylight, sunlight and LEDs, with the blue light showing the highest effect. Results also showed that release of calcein was sensitive to wavelength.

Our results reveal potential of LED-sensitive liposomes for medical and cosmetic applications and that such system can be combined with phototherapy. Such concomitant therapies can increase medical/cosmetic effects and decrease adverse reactions to phototherapy.

## Introduction

Liposomal drug delivery has been studied for decades and in spite of wide application and advantages (1-3), their spatially and temporally drug release behavior is remained to be a major challenge for precise and responsive drug delivery. Triggered controlled drug delivery systems can be used for such purposes (4-6). As a drug-delivery approach, trigger-release liposomes can offer sophisticated targeting and greater control-release capabilities, that provide a greater degree of control over time and place of the drug action (7). Different conditions and energy sources can be used for triggering control release including pH (8, 9), magnetic fields (10, 11), ultrasound (12, 13) and light (14, 15). Some of these triggers, such as light, which is the subject of the present investigation, have therapeutic applications as well (16, 17), like phototherapy and photodynamic therapy (18-22).

Light-sensitive nanoliposomes have been under investigation for years and several release mechanisms have been studied. Different light sources can be used for the above-mentioned applications such as laser, LED (Light Emitting Diode), UV, near –infrared (NIR), and fluorescent lamps (23-30). Among these, and based on the subject of the present investigation, laser and LED are discussed here. Laser light is a single wavelength beam at a high intensity and LED has a narrow range of wavelength, lower intensity and is safer. Both light sources are used in different areas of the body, including the eyes and skin (31-37) and are used for cosmetic applications such as skin rejuvenation, acne treatment, and hyper-pigmented spot removal (38-44). In comparison to laser, LED is much safer and more user friendly. Teuschl *et al.*, studied the healing effects of blue and red LED lights on different type of cells in an *in-vitro* scratch-wound model and showed that these effects depend on the wavelength (18). 

In terms of liposomal drug delivery, laser has been used extensively for triggering liposomes using photo-responsive compounds such as plasmalogen, bis-sorb PC, and azobenzene (45-47). However, there is not much data on application of LED in liposomal drug delivery. Of the rare studies in this area, Bisby *et al.*, showed that blue LED is able to trigger the release of cargo from Bis-Azo PC coating liposomes that works through photo-isomerization (48).

**Figure 1:**
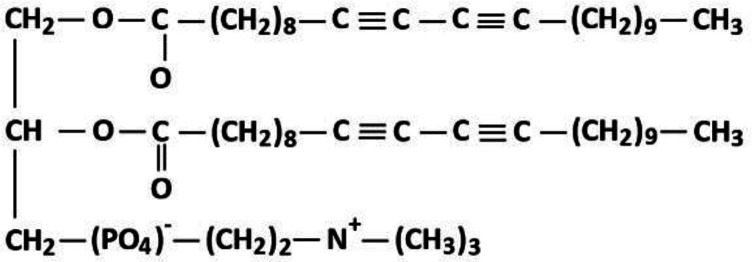
*(1,2 bis(tricosa-10,12-diynoyl)-sn-glycero-3-phosphocholine (DC*_8,9_ *PC) molecular structure*

In the present investigation, we introduce application of LED light with different wavelengths in trigger release of drugs from the liposomes using photo-polymerization mechanisms provided by (1,2 bis(tricosa-10,12-diynoyl)-sn-glycero-3-phosphocholine (DC_8,9_PC). DC_8,9_PC is a diacetylene lipid (Figure 1) and has two diacetylenic hydro-carbon chains which in exposure to light, can bind with maximum 4 nearby DC_8,9_PC molecules to make a polymeric cross-linked network. DC_8,9_PC polymerization have been studied and was concluded that it only occurs when the matrix lipid has a liquid condensed phase in the monolayer or a gel phase in the bilayer liposomes (49), therefore closely packed DPPC matrices have been chosen for the current study.

## Experimental

*Materials*

1,2-dipalmitoyl-sn-glycero-3-phosphocholine (DPPC, > 99.8%), and 1,2-distearoyl -sn-glycero-3-phosphoethanolamine-N-[methoxy(polyethylene glycol)-2000 (DSPE-PEG2000, >98.8%) were purchased from Lipoid GmbH (USA), (1,2 bis(tricosa-10, 12-diynoyl)-sn-glycero-3-phosphocholine (DC_8,9_PC, > 99%) were purchased from Avanti Polar (USA), calcein and HEPES were purchased from Sigma (Germany), cobalt chloride (CoCl_2 _. 6H_2_O), methanol and chloroform were purchased from Merck (Germany). All other materials were of pharmaceutical grade.

*Preparation of liposomes*

Light-sensitive calcein-loaded liposomes were prepared and used in the investigation. For this purpose, a liposomal formulation containing DPPC (86 mol%), DC_8,9_PC (10 mol%), and DSPE-PEG2000 (4 mol%) reported by Yavlovich and coworkers (50), was chosen here. They used this system as laser-sensitive liposomes (not LED). For the present study, the system was loaded with 0.1 mM calcein as described below. DC_8,9_PC is the light sensitive moiety and DSPE-PEG2000 increase the stability of liposomes *in-vitro* (50). Increased stability is expected to prevent unwanted drug release in the absence of the trigger, an important factor for triggered-release systems including our LED-responsive liposomes.

Nanoliposomes were prepared by a lipid film hydration method. Probe sonication was used to reduce their size and increase their uniformity. To perform this method, lipids (DPPC/DC_8,9_PC/DSPE-PEG-2000) were diss-olved in chloroform:methanol mixture (2:1) in an amber vial. The organic solvent was then evaporated at room temperature under nitrogen gas flow while the vial was rotated. To remove trace amount of solvent, the vial was kept under vacuum desiccator overnight. 1ml of HEPES buffer (10 mM HEPES, 140 mM NaCl, pH 7.5) containing 1 mM calcein was then added to the lipid film and the mixture was sonicated by probe sonicator (Ultrasonic Technology Development Co., Iran) for 5 min (10 sec on,10 sec off, 376 W).

Liposomes were then characterized for size, uniformity, and zeta potential. Images of liposomes was also studied using Atomic Force Microscopy (AFM) and Cytation Imaging Reader as described below.

*Size and zeta potential*

Zetasizer Nano ZS ZEN3600 (Malvern Co., England) was used to evaluate the zeta potential, particle size and polydispersity index (PDI) of the prepared nanoliposomes at room temperature. Sizing technique is by dynamic light scattering (DLS) and zeta potential uses laser doppler velocimetry (LDV). Nanoliposomes were diluted in distilled water (1:9), then poured in quartz cell and their size and zeta potential were evaluated.

*Quenching of free calcein*

To discriminate between free and entrapped calcein, CoCl_2_.6H_2_O was added to the prepared liposomes to quench the free calcein (51). Cobalt chloride does not enter liposomes and remain in the external medium (52), therefore, only the released calcein after liposomal breakdown is quenched. In the case liposomes breakdown, the released calcein will all be quenched. For this purpose, 10 µl 40 mM CoCl_2_ was added to the prepared nanoliposomes in HEPES buffer (5:95) and vortexed for 30 seconds.

*Imaging*

To evaluate the morphology of liposomes, the system was studied by imaging using AFM and cytation imaging reader. AFM images were taken with the Nanowizard II AFM, with the intermittent mode manufactured by JPK Company (Germany). 50-100 µL of the prepared nanoliposomes were spread on a microscope slide, dried at room temperature and AFM images were then obtained at the same temperature.

The fluorescence images of the nanoliposomes were taken using the Cytation Imaging Reader (Cytation III) with the GEM5 software (BioTEK, USA). This technique showed calcein loaded nanoliposomes as green spots. This technique uses λ_Em_ and λ_Ex_ of the probe, which were adjusted to be 511 and 485 nm, respectively for calcein. λ_Em_ and λ_Ex _at experimental conditions were measured here using f-2500 Fluorescence Spectrophotometer (HITACHI, Japan).

## Release studies

Release studies at different light conditions were performed using blue, green, red and white (blue+green+red) LEDs (Table 1), as described below.

Release of calcein from liposomes under different LED light wavelengths was investigated at room temperature. The quenched nanoliposome mixture split into several vials and each vial was exposed to a specific condition. The conditions included amber vial in darkness and transparent vial exposed to sunlight, daylight in laboratory, white LED light, blue LED light, green LED light and red LED light as shown in Table 1. To measure the maximum possible calcein release, liposomes were broken by methanol to release all the cargo. Methanol was added to the liposomes, and the resulting mixtures were vortexed for 5 min to lysis them. Our study also showed that water and methanol does not affect fluorescence intensity of calcein. Exposure time of the liposome to LED/lights was chosen to be 5 min, as it has been stated that longer exposure times might end to unwanted reactions (53). Cytation imaging reader was used to measure the fluorescence intensity of the samples as discussed later. Each experiment was repeated for three times.

*Calcein measurement*

The amount of calcein in the sample was measured by a fluorescence method. Calibration curve of calcein dissolved in HEPES buffer was obtained by plotting fluorescence intensity vs. calcein concentration using cytation imaging reader. A 0.1 M stock solution of calcein in HEPES buffer (10 mM HEPES, 140 mM NaCl, pH 7.5) was prepared and calibration curve was drawn up over 10^-9^ to 10^-7^ mM calcein in HEPES buffer using λ_Em_ and λ_Ex _of 511 and 485 nm, respectively. All points were measured in triplicates, and the experiment was performed at room temperature.

*LED source*

Light emitting diode (LED) is a semiconductor light source that emits light when current flows through it. The LED used in the present study was manufactured and validated in our laboratory at Shahid Beheshti University of Medical Sciences. Wavelengths and intensities were validate using UV-Vis spectrometer (190-850 nm) EPP2000 StellarNet Co. (USA) and luxmeter MS6610 MASTECH Co. (USA).

## Results and Discussion

*Characterization of liposomes*

**Figure 2.**
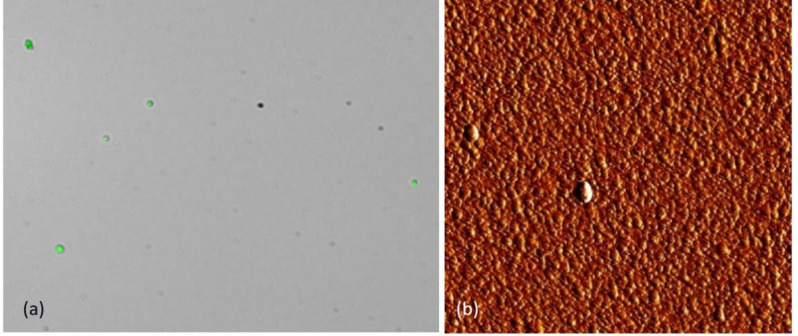
*The fluorescence (a) and AFM (b) images of nanoliposomes at room temperature. AFM image shows uniform spherical particles and green spots in fluorescence image are calcein loaded nanoliposomes*

Figure 2 shows the fluorescence image of calcein loaded liposomes at room temperature (a) and AFM (Atomic Force Microscope) image of the liposomal formulation (b). The fluorescence image shows round hollow nanosize particles containing fluorescence agent as green spots; and AFM image indicate uniform spherical particles. The particle size of liposomes was estimated to be around 100 nm by AFM.

**Figure 3:**
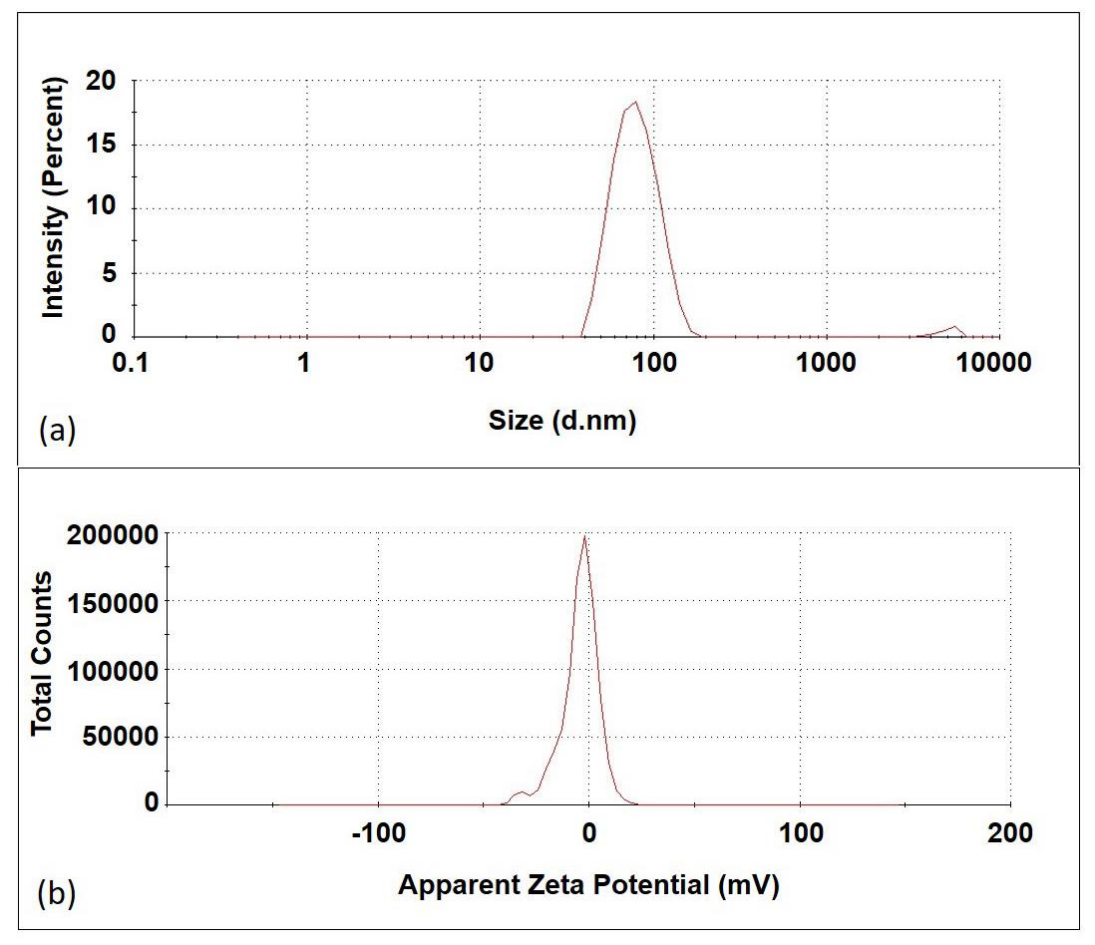
*Size distribution and zeta potential of liposomal formulation; nanoliposome size is 83.87 ± 8.49 nm (mean ± SD, n=3) and zeta potential is -4.66 ± 0.7 mV (mean ± SD, n = 3).*

Figure 3 shows particle size and zeta potential of prepared liposomes obtained by nanozetasizer. Liposomes showed a partially negative zeta potential of -4.66 ± 0.7 mV (mean ± SD, n = 3). In term of size, liposomes showed a particle size of 83.87 ± 8.49 nm (mean ± SD, n = 3) in good agreement with AFM results. Their PDI was 0.163, which represent a good uniformity, in good agreement with imaging results.

*Calcein calibration curve*

**Figure 4:**
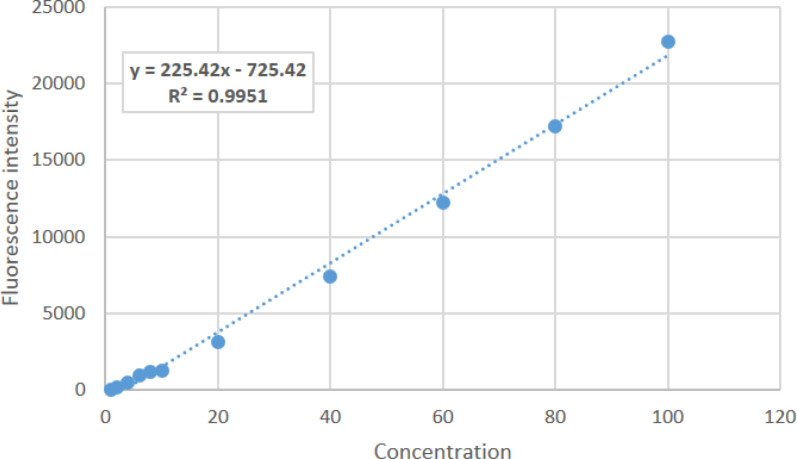
*Calcein calibration curve at pH 7.5 using cytation imaging reader at room temperature*

Calibration curve was drawn up from 10^-9^ to 10^-7^ mM calcein using 11 concentrations at pH 7.5 (Figure 4). Calcein concentration vs. intensity showed a good linear relationship (R^2^ = 0.995) over the studied range (Figure 4).

*Release of calcein from liposomes*

**Figure 5:**
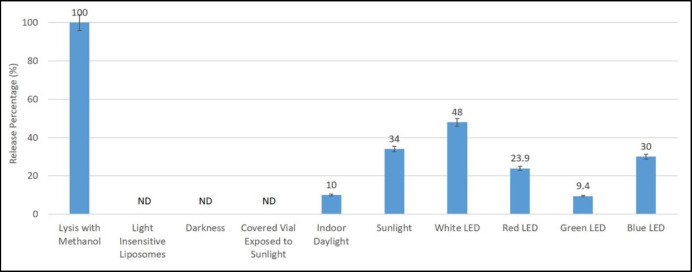
*Comparison of release percentage of the nanoliposome formulation exposed to various conditions and LEDs at room temperature. Lysis with methanol was considered as 100%. Data are mean ± SD (n=3). ND: Not Detected*

Figure 5 shows calcein release from liposomes in several conditions including lysis with methanol, placing in darkness and exposing to the indoor daylight, sunlight, sunlight exposure using vials covered with an opaque film (to study the thermal effect of sunlight), white LED light, red LED light, green LED light and blue LED light. In this study, liposome lysis by methanol which have the maximum release, was considered as 100% release and other liposomal formulations were evaluated in comparison to this value.

According to the results shown in Figure 5, liposomes, placed in darkness (at room temperature) and opaque vials exposed to sunlight showed no significant release over time, while exposing to the indoor daylight and direct sunlight led to up to 10 and 34% release, respectively. White LED with approximately 50% release, showed the highest effect (Figure 5). Light insensitive liposomal formulation (without DC_8,9_PC) also did not show any calcein release. Statistical analysis showed that the difference between sunlight and indoor daylight (*P*-value = 0.000) and white LED and sunlight (*P*-value = 0.021) are significant. These results show that the DC_8,9_PC liposomes release their cargo only in the presence of light and that the effect of light is not a thermal effect. These results indicate that liposomes should be kept in darkness to prevent any possible release due to light from other sources such as daylight. Therefore, such systems require special packaging. However, heating by sunlight did not result in release of the cargo and no protection is required in this regard.

DC_8,9_PC is a lipidic monomer which will be polymerized when exposed to the light as demonstrated by Yavlovich, *et al.* (54), using DC_8,9_PC-containing liposomes exposed to UV laser radiation for up to 45 min. Application of LED in our system provides some advantages. In comparison to laser, LED is much safer and more user friendly with lower cost (55, 56). Besides this, the LED used in the present investigation is in the wavelength range of visible light that is safer than UV.

It worth to note that Kenaan *et al.* (53) studied DC_8,9_PC reactions with UV irradiation and showed that exposing DC_8,9_PC liposomes to UV radiation could result in oxidative reactions and formation of products such as ketones, aldehydes and alcohols, in a time-dependent manner and if they are exposed to UV for more than 8 min. Accordingly, and in spite of the fact that LED is expected to be safer than UV, the radiation time in the present investigation was chosen to be 5 min to reduce such a risk. However, further studies are required to assess reactions of these lipids with LED lights.

Our results show that light-triggered calcein release from liposomes depends on light type and intensity. Exposure to sunlight showed higher release than indoor daylight (10% vs 34%). Intensity of sunlight is about 2000 times of that of indoor daylight. Considering our results (Figure 5) and light properties (Table 1), we can conclude that the sensitivity of liposomes to light more depends on the type (wavelength) of the light than to intensity. This is in good agreement with Leung and Romanowski who suggested that wavelength is the most affecting factor on cargo release from light-sensitive liposomes (57). Each wavelength has a specific effect on a specific compound and, therefore, specific release percentage. Therefore, we cannot expect full release for all wavelengths, even by increasing the intensity. This will explain the small difference in calcein release between sunlight and indoor-daylight, in spite of huge difference between their intensities.

White light contains all the wavelengths in the visible area. There are some reports about different effects of various wavelengths on biological materials. P. Moore, *et al.* investigated the effect of wavelength on proliferation of cultured murine cells and concluded that both wavelength and cell type influence the cell proliferation response to low intensity laser irradiation (58). It has also been shown that different doses and wavelengths of low-level laser therapy (LLLT) show different effects on cytochrome-c oxidase activity in intact skeletal muscle of rats (59). Wang *et al.* also reported that photobiomodulation of human adipose-derived stem cells under different wavelengths operates via different mechanisms of action (60). However, there is no systematic study on the effects of wavelength of light on the release of cargos from light-sensitive liposomes and to the best of our knowledge, reported experiments in the literatures usually deal with only one wavelength (57, 61, 62).

To determine which part of white LED shows more effect, we studied several wavelengths (red, green and blue) in the present investigation. These wavelengths are used for clinical and cosmetic applications. For instances, red range of wavelengths is used in wrinkles, scars, and persistent wounds treatments, green range of wavelengths is used for skin calming, anti-aging and increasing the collagen in the skin and blue range of wavelengths is used for treatment of acne vulgaris, combination therapy and seasonal affective disorders (63). 

Our findings indicate that different wavelengths show different effects on calcein release from liposomes. The highest release was observed for blue LED with about 30% calcein release, followed by 24% release by red LED (Figure 5). Green LED showed lowest amount of calcein release (less than 10%) (Figure 5). Statistical analysis show that the differences between all light LEDs (blue, green and red) or these lights with white LED, are significant (*P*-value < 0.003). Their effects on release are different and red LED with the lowest intensity was more effective than green light, by about 2.5 times. These data are in good agreement with Leung and Romanowski who have suggested that wavelength is the most affecting factor on cargo release from light-sensitive liposomes (57).

*.*

**Table 1:** Characteristics of the LEDs used in the current study

**LED light **	**Wavelength (nm)** ^a^	**Intensities (lx, in 5 cm distance)**
Blue	452	53200
Green	511	40700
Red	630	17400
White (blue+green+red)	452 + 511 + 630	83600
Sunlight		99300
Indoor daylight^b^		44

^a^ LEDs have narrow range of wavelengths which center at a specific number, shown in this table.

^b^ Indoor daylight, is the indirect light in the lab while lamps are turned off.

## Conclusion

The present investigation introduces a LED sensitive liposome that works through photopolymerization, as a novel responsive drug delivery system. LED is widely employed in phototherapy and combination of this technique with sensitive liposomal systems can provide several benefits such as drug therapy for healing during phototherapy. Different agents such as healing agents, peptides, and anti-inflammatory drugs that can improve photo-therapy or decrease its side effects, can be loaded in such liposomes. LED-sensitive liposomes can be used for other applications as well such as responsive topical drug delivery systems or delivery of different agents for cosmetic purposes such as anti-wrinkle peptides. Our results also show that the sensitivity of liposomes to LED is type (wavelength) dependent. Such a property allows adjustment of the treatment through controlling the release of the cargo by using proper light. Our results also show that such liposomes should be kept in darkness to prevent any possible release due to light from other sources such as daylight. Therefore, such systems require special packaging. However, heating by daylight does not result in release of the cargo. Further studies are required to fully explain this area of drug delivery.

## References

[1] Saffari M, Moghimi HR, Dass CR (2016). Barriers to liposomal gene delivery: from application site to the target. Iran. J. Pharm Res..

[2] Movassaghian S, Moghimi HR, Shirazi FH, Koshkaryev A, Trivedi MS, Torchilin VP (2013). Efficient down-regulation of PKC-α gene expression in A549 lung cancer cells mediated by antisense oligodeoxynucleotides in dendrosomes. Int. J. Pharm..

[3] Alinaghi A, Rouini M, Daha FJ, Moghimi H (2014). The influence of lipid composition and surface charge on biodistribution of intact liposomes releasing from hydrogel-embedded vesicles. Int. J. Pharm..

[4] Luo D, Carter KA, Razi A, Geng J, Shao S, Giraldo D, Sunar U, Ortega J, Lovell JF (2016). Doxorubicin encapsulated in stealth liposomes conferred with light-triggered drug release. Biomaterials.

[5] Kono K, Takashima M, Yuba E, Harada A, Hiramatsu Y, Kitagawa H, Otani T, Maruyama K, Aoshima S (2015). Multifunctional liposomes having target specificity, temperature-triggered release, and near-infrared fluorescence imaging for tumor-specific chemotherapy. J. Control. Release.

[6] Wu G, Mikhailovsky A, Khant HA, Fu C, Chiu W, Zasadzinski JA (2008). Remotely triggered liposome release by near-infrared light absorption via hollow gold nanoshells. J. Am. Chem. Soc..

[7] Reimhult E (2015). Nanoparticle-triggered release from lipid membrane vesicles. New Biotechnol..

[8] Zhao Y, Ren W, Zhong T, Zhang S, Huang D, Guo Y, Yao X, Wang C, Zhang W-Q, Zhang X (2016). Tumor-specific pH-responsive peptide-modified pH-sensitive liposomes containing doxorubicin for enhancing glioma targeting and anti-tumor activity. J. Control. Release.

[9] Jiang L, Li L, He X, Yi Q, He B, Cao J, Pan W, Gu Z (2015). Overcoming drug-resistant lung cancer by paclitaxel loaded dual-functional liposomes with mitochondria targeting and pH-response. Biomaterials..

[10] Guo H, Chen W, Sun X, Liu Y-N, Li J, Wang J (2015). Theranostic magnetoliposomes coated by carboxymethyl dextran with controlled release by low-frequency alternating magnetic field. Carbohydr. Polym..

[11] Rodzinski A, Guduru R, Liang P, Hadjikhani A, Stewart T, Stimphil E, Runowicz C, Cote R, Altman N, Datar R (2016). Targeted and controlled anticancer drug delivery and release with magnetoelectric nanoparticles. Sci. Rep..

[12] Crasto GJ, Kartner N, Reznik N, Spatafora MV, Chen H, Williams R, Burns PN, Clokie C, Manolson MF, Peel SA (2016). Controlled bone formation using ultrasound-triggered release of BMP-2 from liposomes. J. Control. Release.

[13] Klibanov A, Du Z, Diakova G (2015). Ultrasound-triggered tumor therapy with doxorubicin-liposome-microbubble complexes in a subcutaneous murine colon adenocarcinoma model. J. Ther. Ultrasound.

[14] Lajunen T, Nurmi R, Kontturi L, Viitala L, Yliperttula M, Murtomäki L, Urtti A (2016). Light activated liposomes: functionality and prospects in ocular drug delivery. J. Control. Release.

[15] Huu VAN, Luo J, Zhu J, Zhu J, Patel S, Boone A, Mahmoud E, McFearin C, Olejniczak J, de Gracia Lux C (2015). Light-responsive nanoparticle depot to control release of a small molecule angiogenesis inhibitor in the posterior segment of the eye. J. Control. Release.

[16] Garza Z, Born M, Hilbers P, Liebmann J (2018). Visible Light Therapy: Molecular Mechanisms and Therapeutic Opportunities. Curr. Med. Chem..

[17] Wang Z, Tang X, Wang X, Yang D, Yang C, Lou Y, Chen J, He N (2016). Near-infrared light-induced dissociation of zeolitic imidazole framework-8 (ZIF-8) with encapsulated CuS nanoparticles and their application as a therapeutic nanoplatform. Chem. Comm..

[18] Teuschl A, Balmayor ER, Redl H, van Griensven M, Dungel P (2015). Phototherapy with LED light modulates healing processes in an in-vitro scratch-wound model using 3 different cell types. Dermatol. Surg..

[19] Zhang H, Liu S, Yang X, Chen N, Pang F, Chen Z, Wang T, Zhou J, Ren F, Xu X (2017). LED Phototherapy With Gelatin Sponge Promotes Wound Healing in Mice. Photochem. Photobiol..

[20] Ebbesen F, Madsen PH, Vandborg PK, Jakobsen LH, Trydal T, Vreman HJ (2016). Bilirubin isomer distribution in jaundiced neonates during phototherapy with LED light centered at 497 nm (turquoise) vs 459 nm (blue). Pediatr. Res..

[21] Asnaashari M, Mojahedi SM, Asadi Z, Azari-Marhabi S, Maleki A (2016). A comparison of the antibacterial activity of the two methods of photodynamic therapy (using diode laser 810 nm and LED lamp 630 nm) against Enterococcus faecalis in extracted human anterior teeth. Photodiagn. Photodyn..

[22] Dong Y, Zhou G, Chen J, Shen L, Jianxin Z, Xu Q, Zhu Y (2016). A new LED device used for photodynamic therapy in treatment of moderate to severe acne vulgaris. Photodiagn. Photodyn..

[23] Huang Y, Yu L, Ren J, Gu B, Longstaff C, Hughes AD, Thom SA, Xu XY, Chen R (2019). An activated-platelet-sensitive nanocarrier enables targeted delivery of tissue plasminogen activator for effective thrombolytic therapy. J. Control. Release.

[24] Oliveira-Silva T, Suzuki L, Kato I, Deana A, Ribeiro M, Prates R (2016). In-vitro Effect Photodynamic Therapy With Led And Methylene Blue On Candida Albicans Pretreated With Glucose. Lasers Surg. Med..

[25] Haak C, Togsverd-Bo K, Thaysen-Petersen D, Wulf H, Paasch U, Anderson R, Haedersdal M (2015). Fractional laser-mediated photodynamic therapy of high-risk basal cell carcinomas–a randomized clinical trial. Brit. J. Dermatol..

[26] Morries LD, Cassano P, Henderson TA (2015). Treatments for traumatic brain injury with emphasis on transcranial near-infrared laser phototherapy. Neuropsych. Dis. Treat..

[27] Maharoof M, Khan SA, Saldanha PR, Mohamed R (2017). Comparison of light emitting diode and compact fluorescent lamp phototherapy in treatment of neonatal hyperbilirubinemia. Int. J. Contemp. Pediatr..

[28] Fang J, Liao L, Yin H, Nakamura H, Subr V, Ulbrich K, Maeda H (2015). Photodynamic therapy and imaging based on tumor-targeted nanoprobe, polymer-conjugated zinc protoporphyrin. Future Sci. OA..

[29] Henderson TA, Morries LD (2015). Near-infrared photonic energy penetration: can infrared phototherapy effectively reach the human brain? Neuropsych. Dis. Treat..

[30] Mreihil K, Madsen P, Nakstad B, Benth JŠ, Ebbesen F, Hansen TWR (2015). Early formation of bilirubin isomers during phototherapy for neonatal jaundice: effects of single vs double fluorescent lamps vs. photodiodes. Pediatr. Res..

[31] Figurová M, Ledecký V, Karasová M, Hluchý M, Trbolová A, Capík I, Horňák S, Reichel P, Bjordal JM, Gál P (2016). Histological assessment of a combined low-level laser/light-emitting diode therapy (685 nm/470 nm) for sutured skin incisions in a porcine model: a short report. Photomed. Laser Surg..

[32] Mamalis A, Koo E, Isseroff RR, Murphy W, Jagdeo J (2015). Resveratrol prevents high fluence red light-emitting diode reactive oxygen species-mediated photoinhibition of human skin fibroblast migration. PloS One..

[33] Li Y, Zhang J, Xu Y, Han Y, Jiang B, Huang L, Zhu H, Xu Y, Yang W, Qin C (2016). The Histopathological Investigation of Red and Blue Light Emitting Diode on Treating Skin Wounds in Japanese Big-Ear White Rabbit. PloS One.

[34] Zhang L, Zhu X, Wang X, Li J, Gao F, Zhou G (2016). Green light-emitting diodes light stimuli during incubation enhances posthatch growth without disrupting normal eye development of broiler embryos and hatchlings. Asian. Austral. J. Anim..

[35] Sahni J, Czanner G, Gutu T, Taylor S, Bennett K, Wuerger S, Grierson I, Murray-Dunning C, Holland M, Harding S (2017). Safety and acceptability of an organic light-emitting diode sleep mask as a potential therapy for retinal disease. Eye.

[36] Ventura BV, Al-Mohtaseb Z, Wang L, Koch DD, Weikert MP (2015). Repeatability and comparability of corneal power and corneal astigmatism obtained from a point-source color light–emitting diode topographer, a Placido-based corneal topographer, and a low-coherence reflectometer. J. Cataract. Refr. Surg..

[37] Scholz P, Altay L, Fauser S (2016). Comparison of subthreshold micropulse laser (577 nm) treatment and half-dose photodynamic therapy in patients with chronic central serous chorioretinopathy. Eye.

[38] Opel DR, Hagstrom E, Pace AK, Sisto K, Hirano-ALi SA, Desai S, Swan J (2015). Light-emitting diodes: a brief review and clinical experience. J. Clin. Aesthet Dermatol..

[39] Baker A (2016). Light-emitting diode red light therapy: evidence base for aesthetic indications. J. Aesthet.Nurs..

[40] Yeh N, Ding TJ, Yeh P (2015). Light-emitting diodes׳ light qualities and their corresponding scientific applications. Renew. Sust. Energ. Rev..

[41] Owen WG, Lopez RG (2015). End-of-production supplemental lighting with red and blue light-emitting diodes (LEDs) influences red pigmentation of four lettuce varieties. HortScience.

[42] Filippini M, Del Duca E, Negosanti F, Bonciani D, Negosanti L, Sannino M, Cannarozzo G, Nistico SP (2017). Fractional CO2 laser: from skin rejuvenation to vulvo-vaginal reshaping. Photomed. Laser. Surg..

[43] Brauer JA, Kazlouskaya V, Alabdulrazzaq H, Bae YS, Bernstein LJ, Anolik R, Heller PA, Geronemus RG (2015). Use of a picosecond pulse duration laser with specialized optic for treatment of facial acne scarring. JAMA Dermatol..

[44] Passeron T, Genedy R, Salah L, Fusade T, Kositratna G, Laubach HJ, Marini L, Badawi A (2019). Laser treatment of hyperpigmented lesions: position statement of the European Society of Laser in Dermatology. J. Eur. Acad. Dermatol..

[45] Luo D, Li N, Carter KA, Lin C, Geng J, Shao S, Huang WC, Qin Y, Atilla-Gokcumen GE, Lovell JF (2016). Rapid light-triggered drug release in liposomes containing small amounts of unsaturated and porphyrin–phospholipids. Small.

[46] Sharma NK, Kumar V, Augustine R, Kalarikkal N, Oluwafemi OS, Joshy KS, Thomas S (2016). Triggerable liposomes: newer approach in cytoplasmic drug delivery. Nanomedicine and Tissue Engineering.

[47] Yao C, Wang P, Li X, Hu X, Hou J, Wang L, Zhang F (2016). Near-Infrared-Triggered Azobenzene--Liposome/Upconversion Nanoparticle Hybrid Vesicles for Remotely Controlled Drug Delivery to Overcome Cancer Multidrug Resistance. Adv. Mater..

[48] Bisby RH, Mead C, Morgan CG (2000). Wavelength-programmed solute release from photosensitive liposomes. Biochem. Bioph. Res. Co..

[49] Callens M, Beltrami M, D’Agostino E, Pfeiffer H, Verellen D, Paradossi G, Van Den Abeele K (2019). The photopolymerization of DC8, 9PC in microbubbles. Colloid. Surf. A: Physicochem. Eng. Asps..

[50] Yavlovich A, Singh A, Blumenthal R, Puri A (2011). A novel class of photo-triggerable liposomes containing DPPC: DC8, 9PC as vehicles for delivery of doxorubcin to cells. BBA-Biomembranes..

[51] Huang S-L, MacDonald RC (2004). Acoustically active liposomes for drug encapsulation and ultrasound-triggered release. BBA-Biomembranes.

[52] Oku N, Kendall Da, MacDonald RC (1982). A simple procedure for the determination of the trapped volume of liposomes. BBA-Biomembranes.

[53] Kenaan A, Cheng J, Qi D, Chen D, Cui D, Song J (2018). Physicochemical analysis of DPPC and photopolymerizable liposomal binary mixture for spatiotemporal drug release. Anal. Chem..

[54] Yavlovich A, Singh A, Tarasov S, Capala J, Blumenthal R, Puri A (2009). Design of liposomes containing photopolymerizable phospholipids for triggered release of contents. J. Therm. Anal. Calorim..

[55] Sliney DH (1997). Laser and LED eye hazards: safety standards. Optics and Photonics News (OPN).

[56] Brancaleon L, Moseley H (2002). Laser and non-laser light sources for photodynamic therapy. Lasers Med. Sci..

[57] Leung SJ, Romanowski M (2012). Light-activated content release from liposomes. Theranostics.

[58] Moore P, Ridgway TD, Higbee RG, Howard EW, Lucroy MD (2005). Effect of wavelength on low-intensity laser irradiation-stimulated cell proliferation in-vitro. Lasers Surg. Med..

[59] Albuquerque-Pontes GM, de Paula Vieira R, Tomazoni SS, Caires CO, Nemeth V, Vanin AA, Santos LA, Pinto HD, Marcos RL, Bjordal JM (2015). Effect of pre-irradiation with different doses, wavelengths, and application intervals of low-level laser therapy on cytochrome c oxidase activity in intact skeletal muscle of rats. Laser. Med. Sci..

[60] Wang Y, Huang Y-Y, Wang Y, Lyu P, Hamblin MR (2017). Photobiomodulation of human adipose-derived stem cells using 810 nm and 980 nm lasers operates via different mechanisms of action. BBA-General Subjects.

[61] Yan F, Duan W, Yekuo Li HW, Zhou Y, Pan M, Liu H, Liu X, Zheng H (2016). NIR-laser-controlled drug release from DOX/IR-780-loaded temperature-sensitive-liposomes for chemo-photothermal syner-gistic tumor therapy. Theranostics.

[62] Wiraja C, Mathiyazhakan M, Movahedi F, Upputuri PK, Cheng Y, Pramanik M, Yang L, Becker DL, Xu C (2016). Near-infrared light-sensitive liposomes for enhanced plasmid DNA transfection. Bioeng. Transl. Med..

[63] Oh P-S, Jeong H-J (2019). Therapeutic application of light emitting diode: Photo-oncomic approach. J. Photoch. Photobio. B..

